# A Novel Compound Heterozygous *CYP17A1* Variant Causes 17α-Hydroxylase/17, 20-Lyase Deficiency

**DOI:** 10.3389/fgene.2019.00996

**Published:** 2019-10-22

**Authors:** Hong Chen, Ke Yuan, Bingtao Zhang, Zexiao Jia, Chun Chen, Yilin Zhu, Yaping Sun, Hui Zhou, Wendong Huang, Li Liang, Qingfeng Yan, Chunlin Wang

**Affiliations:** ^1^Department of Pediatrics, The First Affiliated Hospital, College of Medicine Zhejiang University, Hangzhou, China; ^2^College of Life Science, Zhejiang University, Hangzhou, China; ^3^Department of Diabetes Complications and Metabolism, The Beckman Research Institute, City of Hope National Medical Center, Duarte, CA, United States; ^4^Key Laboratory for Cell and Gene Engineering of Zhejiang Province, Hangzhou, China

**Keywords:** *CYP17A1* gene, 17α-hydroxylase/17,20-lyase deficiency, congenital adrenal hyperplasia, rhabdomyolysis, variant

## Abstract

**Background:** Congenital adrenal hyperplasia (CAH) encompasses a group of autosomal recessive diseases characterized by enzyme deficiencies, within steroid hormone anabolism, which lead to disorders in cortisol synthesis. The 17α-hydroxylase/17,20-lyase deficiency (17-OHD) is an uncommon form of CAH caused by variants in the *CYP17A1* gene.

**Aims:** We report a novel compound heterozygous *CYP17A1* variant and its association with the pathogenesis of 17-OHD.

**Methods:** The patient was assessed for medical history, clinical manifestations, physical examination, laboratory examination, karyotype analysis, and adrenal computed tomography. Mutation screening was conducted using whole-exome sequencing (WES) and Sanger sequencing. The wild-type and mutant *CYP17A1* complementary DNAs (cDNAs) were amplified and cloned into a pcDNA3.1(+) vector. These plasmids were transfected transiently into HEK-293T cells. Quantitative PCR and Western blotting analysis were performed to measure the expression level of P450c17. An enzymatic activity assay was conducted to measure the content of 17-hydroxyprogesterone (17-OHP) and dehydroepiandrosterone (DHEA) in medium using liquid chromatography–tandem mass spectrometry (LC-MS/MS).

**Results:** The proband was characterized by 17-OHD with rhabdomyolysis, hypokalemia, and adrenal insufficiency. Novel compound heterozygous variants of the *CYP17A1* gene (c.1304T > C/p.Phe435Ser and c.1228delG/p.Asp410Ilefs*9) were identified. The enzymatic activity assay revealed that this variant resulted in a complete deficiency of 17α-hydroxylase and 17,20-lyase activity. This was consistent with the hormonal characteristics of the proband’s blood.

**Conclusions:** These results suggest that the compound heterozygous variant of c.1304T > C and c.1228delG of the *CYP17A1* gene can lead to 17-OHD. Our findings thus provide a novel insight into the clinical evaluations and molecular basis of 17-OHD.

## Introduction

Congenital adrenal hyperplasia (CAH) encompasses a group of autosomal recessive diseases characterized by enzyme deficiencies, within steroid hormone anabolism, which lead to disorders in cortisol synthesis. In particular, these genetic defects can hinder the synthesis of glucocorticoids, mineralocorticoids, or sex hormones and lead to the accumulation of upstream products. CAH results in a series of clinical manifestations including insufficient or complete lack of cortisol synthesis, adrenocorticotropic hormone (ACTH) secretion feedback, and increased and stimulated adrenal cortical cell proliferation (Yanase et al., 1991; Turcu and Auchus, 2015).

17α-Hydroxylase/17, 20-Lyase deficiency (17-OHD) [Online Mendelian Inheritance in Man (OMIM) 202110] is a rare type of CAH with a reported incidence of approximately 1/1,000,000 births (Auchus, 2001). 17-OHD is caused by variants in the *CYP17A1* gene, which is located in chromosome 10q24.3. The encoded P450c17 encompasses the activities of both 17α-hydroxylase and 17,20-lyase (Miller, 2012; Kim et al., 2014). During the synthesis of steroid hormones, pregnenolone and progesterone are catalyzed by 17α-hydroxylase, producing 17-hydroxypregnenolone and 17-hydroxyprogesterone (17-OHP), respectively. P450c17 is an important rate-limiting step in the above catalytic process. Two cofactors, P450 oxidoreductase and cytochrome b5, are crucial for P450c17 function by providing electrons or electron flux. The latter crucially enhances 17,20-lyase activity. P450c17 encompasses the activity of 17,20-lyase, which is the only enzyme required for dehydroepiandrosterone (DHEA) synthesis from pregnenolone. Defects in the synthesis of sex steroids result in the appearance of female genitals in both men and women. Adolescent girls usually seek help for delayed puberty. Different mutations in the *CYP17A1* gene result in a series of impaired or reduced functions relating to reduce P450c17 enzyme activity, leading to a range of clinical problems. P450c17 defects or dysfunctions, depending on the severity of functional impairment, show up clinically in varying degrees and in the combinations of 17-OHD and 17,20-lyase deficiency. The diagnosis of 17-OHD is based on clinical manifestations, serum hormone levels, and gene sequencing. In addition, urine steroid profiling has been widely used in many countries, which can greatly facilitate diagnosis. However, the clinical presentation and laboratory testing of this disorder remain highly variable and are related to the activity of the enzyme.

In this study, we elaborated the clinical features, laboratory tests, and gene sequencing of a proband who displayed rhabdomyolysis with adrenal insufficiency at disease onset. A novel compound heterozygous variant (c.1304T > C/p.Phe435Ser and c.1228delG/p.Asp410Ilefs*9) of the *CYP17A1* gene was identified using whole-exome sequencing (WES) and Sanger sequencing. These variants resulted in a complete deficiency of 17α-hydroxylase and 17,20-lyase activity.

## Materials and Methods

### Subjects

The study was approved by the Ethics Committee of The First Affiliated Hospital, Zhejiang University, China (No. 2018-727), and conducted in agreement with the Principles of the Declaration of Helsinki. Written and informed consent was obtained from the parents of the proband for publication of this case report.

### Clinical Evaluations

The proband was assessed for medical history, clinical manifestations, physical examination, laboratory examination, karyotype analysis, and adrenal computed tomography.

### Cytogenetic and Molecular Studies

Blood karyotyping was performed using standard techniques. Genomic DNA was isolated from the whole blood of participants using a TaKaRa blood genome DNA extraction kit (TaKaRa Biotechnology). Each of 100-ng purified DNA was underwent a process of end repair, phosphorylation, and ligation to barcoded sequencing adapters. Ligated DNA was size-selected for lengths between 200 and 350 bp and subjected to exonic hybrid capture. Each of the captured libraries was multiplexed and sequenced on multiple Illumina HiSeq X Ten (Illumina, San Diego, CA, USA) flow cells to average target exome coverage of 100× in neoplastic DNA using 150-bp paired-end reads. WES was performed using the IDT The xGen Exome Research Panel v1.0 full-exon capture chip, which was then sequenced using an Illumina NovaSeq 6000 sequencer. Raw data FASTQ files, produced *via* a sequencing platform, were analyzed, and the sequencing quality was assessed using NextGENe (US SoftGenetics). Variant Call Format (VCF) files were then generated. Variability classification used a three-factor grading system and the American College of Medical Genetics (ACMG) genetic variation grading system.

The candidate causal genes discovered *via* WES were then confirmed by Sanger sequencing. The primers were designed using Primer-BLAST [National Center for Biotechnology Information (NCBI)]. The PCR products were sequenced using an ABI 3730 DNA sequencer (Applied Biosystems, Foster City, CA, USA). Sanger sequencing results were analyzed using Chromas Lite v2.01 (Technelysium Pty Ltd., Tewantin, QLD, Australia). The Sanger sequencing variants detected in the *CYP17A1* gene are described according to the NCBI entry NG_007955.1 (NM_000102.3,NP_000093.1).

Analysis of functional affect and conservation of variants was done using the bioinformatics software PolyPhen-2 (http://genetics.bwh.harvard.edu/pph2/) and MutationTaster (http://www.mutationtaster.org). 

### Molecular Modeling

The three-dimensional structure of human p450c17 [Protein Data Bank (PDB) accession code 3RUK] was used to study the effect of the novel variant p.Phe435Ser on protein conformation (DeVore and Scott, 2012). A structural representation was generated using Swiss-Pdb Viewer program (http://www.expasy.org/spdbv/). The variant image of p.Asp410Ilefs*9 was generated using the Swiss-Model (https://swissmodel.expasy.org/interactive) and PyMOL.

### Construction of *CYP17A1* Variants

The wild-type (WT) full-length human *CYP17A1* complementary DNA (cDNA) (NM_000102.3) with *Hin*dIII and *Bam*HI restriction enzyme sites was chemically synthesized (Tsingke, China) and was cloned into the plasmid vector pcDNA3.1(+). The c.1304T > C/p.Phe435Ser and c.1228delG/p.Asp410Ilefs*9 variants were introduced into the *CYP17A1* sequence using seamless cloning (Tsingke, China). The cDNA was amplified using PCR and variants, and a 15-to-20-bp vector terminal homologous sequence was introduced. The circular plasmid was complementarily paired using the interaction between the bases (Gibson et al., 2010) (**Table S1**). The connected product was transformed into *Escherichia coli* DH5α. Positive clones were picked and sequenced using pcDNA3.1(+) universal primers to confirm site-directed mutagenesis.

### Transient Transfection of *CYP17A1* cDNA

We tested seven groups, including a negative control [with only plasmid pcDNA3.1(+)], WT (with pcDNA3.1-WT-*CYP17A1*), and experimental groups (with pcDNA3.1-mut-*CYP17A1* of c.1304T > C, c.1228delG, c.1304T > C/c.1228delG, WT/c.1304T > C, or WT/c.1228delG). For transient transfection, HEK-293T cells were cultured in 24-well plates until 60–70% confluence. The cells were then transfected with a total of 500-ng DNA using a Lipofectamine™ 3000 transfection reagent (Thermo Fisher Scientific, USA). The DNA ratio was 1:1 (250 ng each) when each different variant was co-transfected.

### Quantitative Real-Time PCR

After transfection for 46 h, cells were collected and total RNA preparations were obtained using TRIzol reagent (Invitrogen). Messenger RNA (mRNA) expression levels were quantified by real-time PCR using an ABI PRISM 7900HT Sequence Detection System (Applied Biosystems) (Liu et al., 2014). The forward and reverse primers used for PCR amplification are shown in **Table S1**.

### Western Blotting

After transfection for 46 h, proteins of the seven groups of cells were extracted using a Whole Cell Extraction Kit (Abcam). Each group was added with 12% SDS-PAGE with 10 µg of protein, then electro-transferred onto a polyvinylidene difluoride membrane, and subjected to western blotting. Western blotting was performed using rabbit monoclonal anti-P450c17 antibody (Abcam, ab125022). Immunoreactive bands were visualized on the membrane using enhanced chemiluminescence reagents (Super Signal, Pierce) and a CLINX ChemiScope and ECL system (CWBIO) (Li et al., 2018).

### Enzymatic Activity Assay

After transfection for 40 h, 2 µmol/L of progesterone (Sigma-Aldrich) and 2 µmol/L of 17α-hydroxypregnenolone (Sigma-Aldrich) were added and incubated. The medium was collected after 6 h of intervention, and the concentrations of 17-OHP and DHEA in the medium were measured using liquid chromatography–tandem mass spectrometry (LC-MS/MS) (Agilent 1290 infinity II/AB5500, Agilent, USA) (Kim et al., 2014; Rubtsov et al., 2015).

### Statistical Analysis

Statistical analysis was carried out using Student’s unpaired, two-tailed *t*-test contained in the GraphPad Prism 7 program. A *P*-value <0.05 was considered statistically significant. All data represent three control individuals (three repeats), with at least three independent experiments. Data are represented as mean ± SD (*n* = 3).

## Results

### Clinical Evaluations

**Figure 1:**
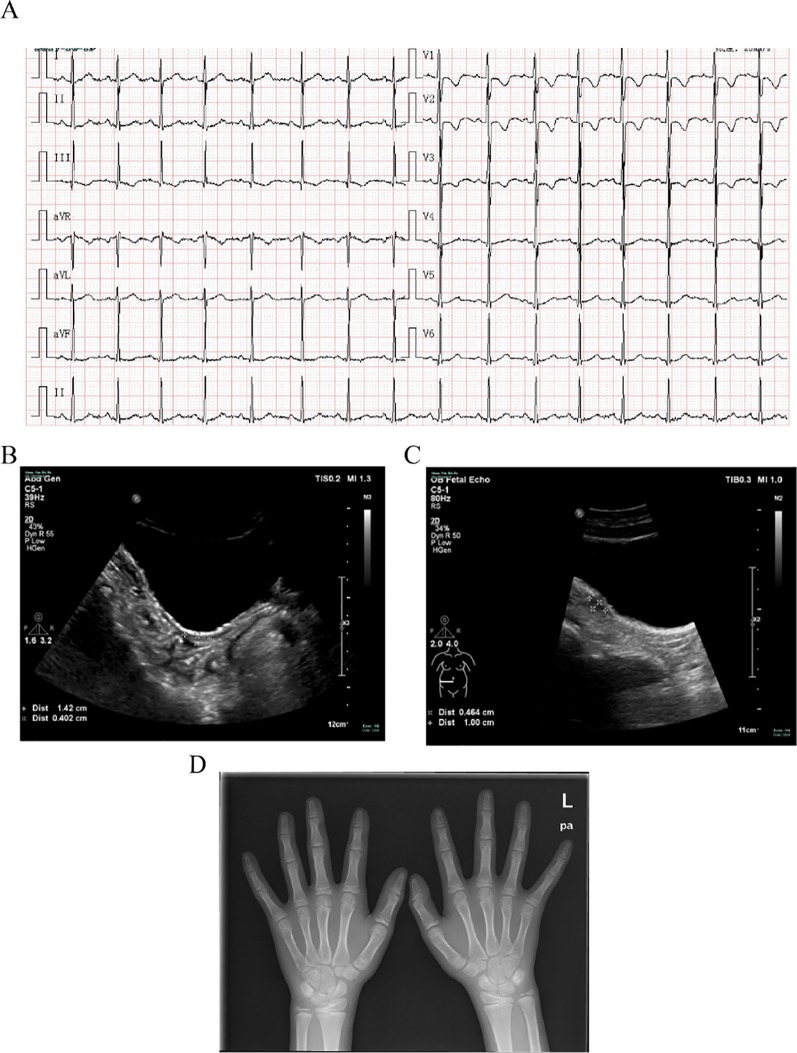
Clinical evaluations. **(A)** Electrocardiogram revealing signs of hypokalemia with sinus tachycardia and ST-T changes. **(B)** Pelvic ultrasound showing that the size of the uterus is about 1.4 cm × 0.4 cm × 0.7 cm. **(C)** Pelvic ultrasound also showing that the size of the suspected right ovary is about 1.0 cm × 0.5 cm × 0.5 cm but the left ovary is undetectable. **(D)** X-ray imaging of the phalanx, carpal, and radioulnar bones. Greulich-and-Pyle methods are used to evaluate bone age.

An 11-year-old girl was admitted to hospital due to rhabdomyolysis without obvious incentives. Her serum biochemical tests revealed rhabdomyolysis, hypokalemia, and metabolic alkalosis (**Table 1**). The blood hormonal characteristics of the proband were also examined. Blood hormone tests revealed a sex steroid deficiency, hypergonadotropic hypogonadism, with low-renin hypertension characterized by low levels of sex hormones and cortisol_8:00am_ and high levels of gonadotropin (**Table 1**). A cosyntropin stimulation test or short synacthen test has not been performed, and early-morning cortisol level was low. After her blood pressure was lowered and fluid and potassium supplementation therapy has been provided, the patient’s myoglobinuria disappeared. After admission, her blood pressure peaked at 138/96 mmHg and then improved. Although she received potassium supplementation therapy, her blood potassium failed to recover. The cause of hypokalemia remained unknown. An electrocardiogram (ECG) revealed signs of hypokalemia with sinus tachycardia and ST-T changes (**Figure 1A**). Pelvic ultrasound showed that there was a prepubertal uterus in shape and appearance with gonads identified as ovaries. The size of the uterus was about 1.4 cm × 0.4 cm × 0.7 cm (**Figure 1B**). The suspected size of the right ovary was about 1.0 cm × 0.5 cm × 0.5 cm, but the left ovary was undetectable (**Figure 1C**). According to the Tanner scale, both her breasts and pubic hair were Tanner 1. Her height was 140 cm (25th centile), and her body weight was 36 kg (25th–50th centiles). Her calculated bone age based on the hand X-ray was of a ∼9-year-old according to the Greulich-and-Pyle method (**Figure 1D**), 2 years younger than her actual age. Her parental heights are 164 and 158 cm. The patient’s genetic height is 154.5 ± 5 cm while her predicted adult height is 169.3 ± 5 cm (> 75th centile). A hyperpigmentation of her skin was found, especially at the joint folds and lip. She had no pubic hairs and no axillary hairs. The karyotype of the proband was shown as normal 46,XX (**Figure S1**). These results strongly suggested that the two enzymatic activities of the proband’s steroidogenic pathway, 17α-hydroxylase and 17,20-lyase, had been impaired. Furthermore, adrenal computed tomography scanning revealed adrenal hyperplasia.

**Table 1:** Blood biochemical and hormonal characteristics of the proband.

Items	Patient	Normal value
Biochemical index		
K^+^	2.90↓	3.50–5.20 mmol/L
AST	573↑	5.0–40.0 U/L
ALT	485↑	5.0–49.0 U/L
LDH	719↑	120–300 U/L
CK	38,400↑	8–60 U/L
CK-MB	747↑	0–25 U/L
Myoglobin	72↑	0.72–4.49 nmol/L
Creatinine	48	15–77 μmol/L
CPK	3,876↑	26–140 U/L
HBDH	580↑	72–300 U/L
HCO_3_^−^	28.7↑	22.0–27.0 mmol/L
Hormones		
ACTH	403.76↑	0.00–90.16 pmol/L
LH	15.89↑	0.33–6.10 IU/L
FSH	43.90↑	1.37–6.97 IU/L
Cortisol_8:00am_	0.06↓	0.22–1.10 μmol/L
PRA	<0.09↓	0.09–5.93 nmol/L/h
Progesterone	7.98↑	1.27–3.82 nmol/L
Aldosterone	115.56↓	168.88–723.78 pmol/L
17-OHP	0.10↓	0.80–1.80 nmol/L
Androstenedione	<1.05↓	1.05–11.57 nmol/L
Estradiol	<43.32↓	136.76–315.00 pmol/L
Testosterone	0.59↓	0.70–1.70 nmol/L
DHEA-S	0.25↓	0.90–15.96 μmol/L

AST, aspartate aminotransferase; ALT, alanine aminotransferase; LDH, lactate dehydrogenase; CK, creatine phosphokinase; CK-MB, creatine kinase muscle/brain; CPK, creatine phosphokinase; HBDH, hydroxybutyrate dehydrogenase; ACTH, adrenocorticotropic hormone; PRA, plasma renin activity; LH, luteinizing hormone; FSH, follicle-stimulating hormone; 17-OHP, 17*α*-hydroxyprogesterone; DHEA-S, dehydroepiandrosterone sulfate.

### Genetic Diagnosis

**Figure 2:**
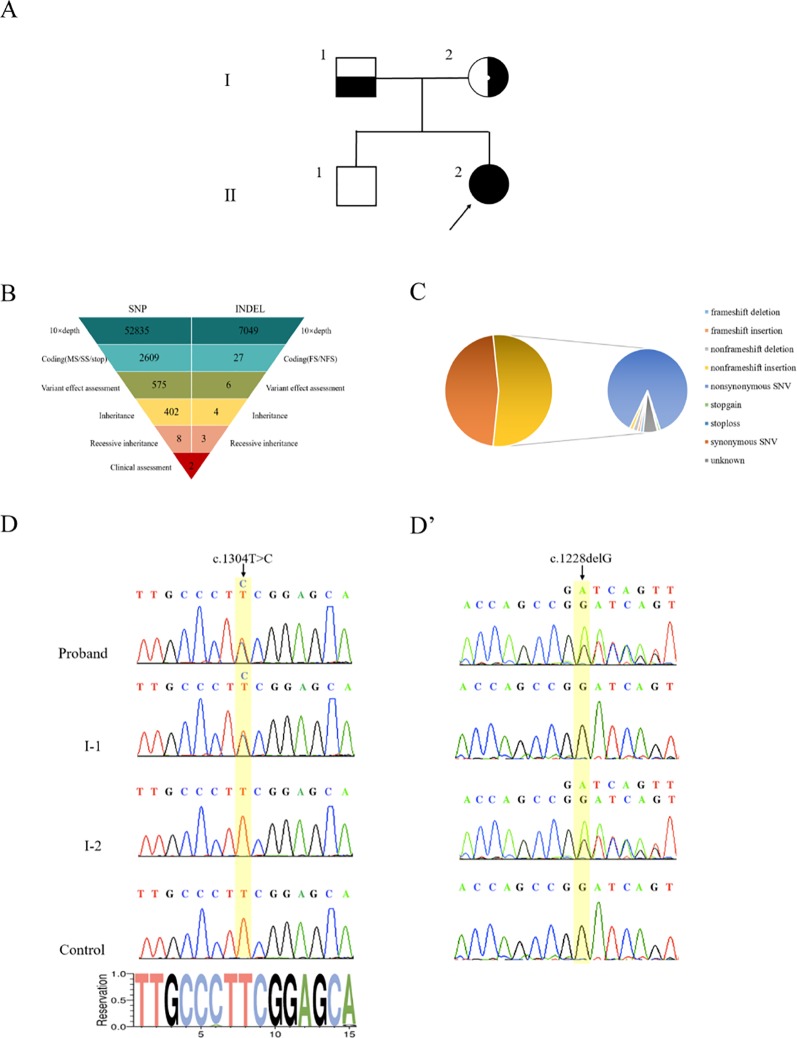
Genetic diagnosis. **(A)** The pedigree with 17α-hydroxylase/17,20-lyase deficiency. The arrowhead denotes the proband. **(B)** Schematic representation of the exome-data-filtering approach under the assumption of recessive inheritance in the family. The following abbreviations are used: MS, missense variant; SS, splice-site variant; stop, stop-codon variant; FS, frameshift indel; and NFS, non-frameshift indel. **(C)** Percentage of variants in exome regions. **(D)** Sanger sequencing chromatograms showing that the proband and her father (I-1) harbor a heterozygous c.1304T > C variant of the *CYP17A1* gene. **(D’)** Sanger sequencing chromatograms showing that the proband and her mother (I-2) harbor a heterozygous c.1228delG deletion of the *CYP17A1* gene.

Genomic DNA was extracted from peripheral blood samples of the proband and her parents (**Figure 2A**). With an average base quality of 40 in FASTQ files and a coverage of 99.59% in targeted regions, the proband’s WES satisfied the mutation screening criteria (**Figures S2A,B**, **Table S2**). There were a total of 26,012 variants in the exome region, of which the proportion of nonsynonymous variants accounted for 53.31%, including missense variants, code shift variants, and fragment insertions and deletions (**Figures 2B,C**). The 13,866 nonsynonymous variants were filtered into 1,127 rare variants according to the mutation frequency data with a minor allele frequency (MAF) of <0.01 or without records in the 1000G_ALL database (http://www.internationalgenome.org/). The correlation degree of phenotype analysis of the remaining sites was determined by “hypokalemia, hypertension and rhabdomyolysis,” where the value of selecting genes was significantly higher than that of other genes. Finally, two loci were concentrated with variants of the *CYP17A1* gene (**Figure 2B**).

PCR products of the *CYP17A1* gene were amplified. Sanger sequencing verified that the proband’s mother carried only the c.1228delG heterozygous deletion without the c.1304T > C variant and the proband’s father carried only the c.1304T > C heterozygous variant without the c.1228delG deletion. The proband carried both c.1304T > C and c.1228delG heterozygous variants of *CYP17A1* (**Figures 2D,D’**). These results indicated that the proband’s two compound variants came from her mother and father, respectively. The two variants were located in the different chromosome. The c.1228delG deletion led to p.Asp410Ilefs*9 codon displacement and production of truncated protein. The c.1304T > C variant resulted in the change of the Phe435 residue to a Ser435 residue. Phe435 is conserved among the P450c17s from different vertebrates (*Pan troglodytes*, *Felis catus*, *Mus musculus*, *Gallus gallus*, *Danio rerio*, and *Xenopus tropicalis*), which suggests that this residue may be critical (**Figure S3**). The PolyPhen-2 software predicted that the p.Phe435Ser variant of the *CYP17A1* gene may be pathogenic, with a probability of a correct prediction of 99.3%, sensitivity of 87%, and a specificity of 96%. MutationTaster predictive analysis also showed that the p.Phe435Ser variant may be pathogenic with a prediction accuracy of 99.9%.

### Three-Dimensional Structure Modeling

**Figure 3:**
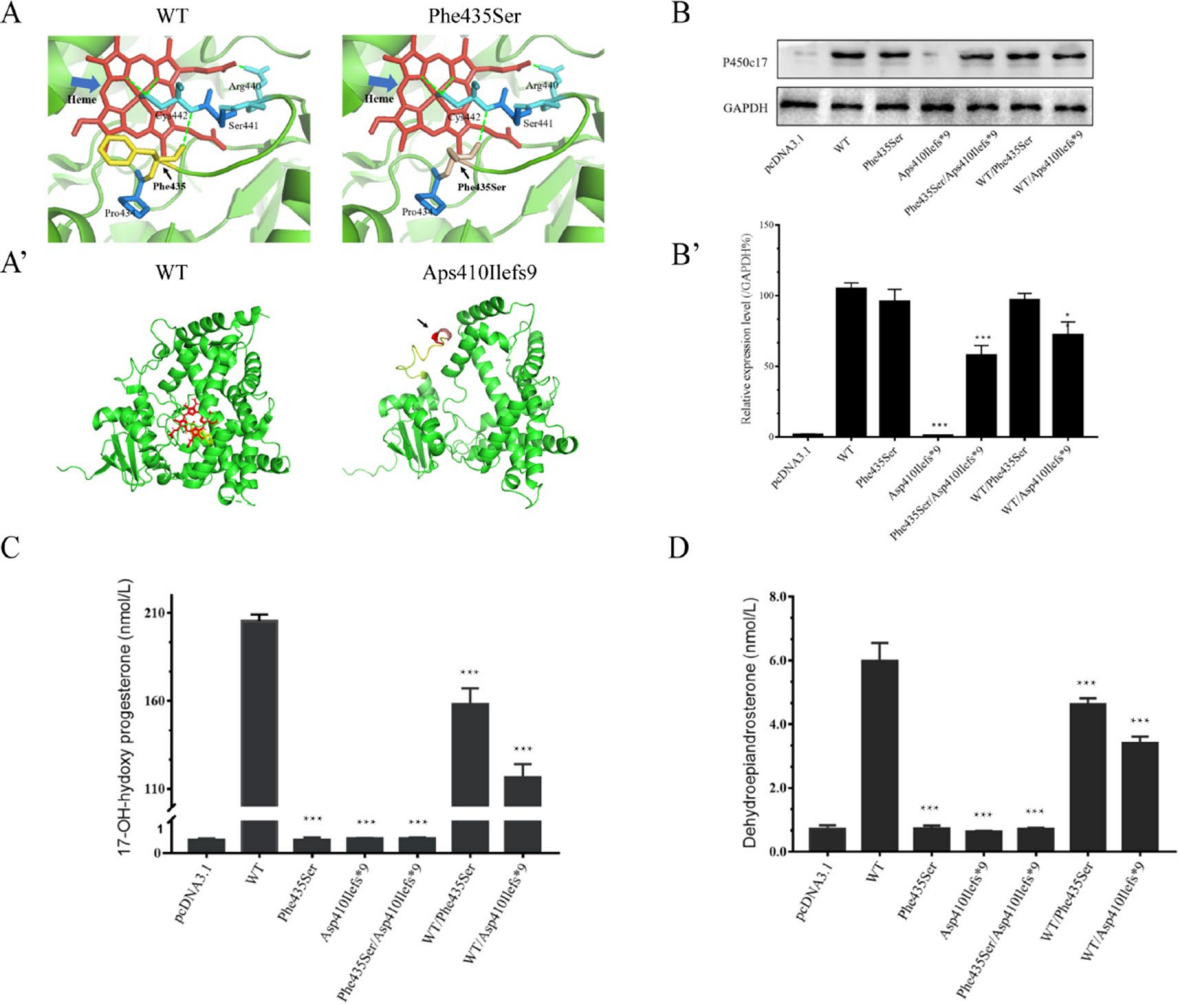
Structural and functional analysis. **(A**, **A’)** Three-dimensional structure as predicted by the Swiss-Pdb Viewer program. **(A)** Phe435 interacts with Cys442 through hydrogen bonds. The conversion of nonpolar phenylalanine into polar serine will disturb the hydrophobic environment. Heme is shown in red, and hydrogen bonding with a green dot. **(A’)** The p.Asp410Ilefs*9 frameshift variant resulting in a truncated protein. **(B)** Western blotting analysis of P450c17 from HEK-293T transfected cell lines. Glyceraldehyde 3-phosphate dehydrogenase (GAPDH) is shown as a loading control. **(B’)** Quantified analysis of Western blotting of P450c17. **(C**, **D)** Enzymatic activity assays of 17-hydroxylase and 17,20-lyase from HEK-293T transfected cell lines. **(C)** The production of 17-hydroxyprogesterone (17-OHP) in the presence of progesterone is indicated as a measurement of 17-hydroxylase activity. **(D)** The production of dehydroepiandrosterone (DHEA) in the presence of 17-hydroxypregnenolone is indicated as a measurement of 17,20-lyase activity. Data are expressed as mean ± standard deviation (SD) (*n* = 3). *p < 0.1, **p < 0.01, ***p < 0.001.

The Swiss-Pdb Viewer program indicated that Phe435 is part of the central hydrophobic cluster which interacts with Cys442, as a heme axial ligand, through hydrogen bonds. Phe435 appears to be located at the C-terminus of the meander peptide and seems to contribute to hydrophobic interactions, helping to stabilize the flap between the heme-binding domain and the meander protein. The conversion of nonpolar phenylalanine into polar serine will disturb the hydrophobic environment, thereby interfering with the catalytic activity and the heme-binding domain (**Figure 3A**). The *CYP17A1* c.1228delG variant led to a p.Asp410Ilefs*9 frameshift variant from amino acid residue 410 and a stop codon in amino acid residue 419, resulting in a truncated protein (**Figure 3A’**).

### Expression Analysis of P450c17

Quantitative PCR results revealed no *CYP17A1* gene differences in mRNA levels among groups (**Figure S4**). Western blotting showed that the translation efficiency of p.Phe435Ser and WT/p.Phe435Ser variants was similar to that of the WT, while the protein band for the p.Asp410Ilefs*9 variant was not imaged at 55 kD. This may result in changes in protein structure or reduced protein stability due to deletion variants. As expected, the protein bands carrying the deletion variants were relatively light. The band of the p.Phe435Ser/p.Asp410Ilefs*9 protein variant was approximately half of that of the WT. The protein band of the WT/p.Asp410Ilefs*9 variant was 68% that of the WT (**Figures 3B,B’**). These results indicated that there was no difference in transcription levels between the two variants and the WT. The difference in the p.Asp410Ilefs*9 variant at the protein level may be due to changes in its three-dimensional structure or protein instability.

### *In Vitro* Functional Analysis of 17α-Hydroxylase and 17,20-Lyase

The 17α-hydroxylase activity was evaluated by measuring the content of 17-OHP in the presence of progesterone in the transfected HEK-293T cells. The content of 17-OHP was 205.35 ± 3.77 nmol/L in the WT (with pcDNA3.1-WT-*CYP17A1*) and 0.41 ± 0.04 nmol/L in the negative control (with only the pcDNA3.1 plasmid). The concentration of 17-OHP was about 158.12 ± 8.95 nmol/L in the WT/p.Phe435Ser heterozygous variant (the proband’s father mutant-like) and 116.55 ± 7.45 nmol/L in the WT/p.Asp410Ilefs*9 heterozygous variant (the proband’s father mutant-like), which had significantly decreased to 77 ± 0.06% and 57 ± 0.03% of the WT, respectively. The content of 17-OHP in the p.Phe435Ser/p.Asp410Ilefs*9 compound heterozygous variants (the proband mutant-like) was 0.45 ± 0.03 nmol/L, which was similar to that of the negative control (**Figure 3C**).

The 17,20-lyase activity was evaluated by measuring the content of DHEA in the presence of 17-hydroxypregnenolone in the transfected HEK-293T cells. The DHEA contents were 5.99 ± 0.56 nmol/L in the WT and 0.71 ± 0.11 nmol/L in the negative control. The DHEA contents were 4.63 ± 0.18 nmol/L in the WT/p.Phe435Ser heterozygous variant and 3.42 ± 0.19 nmol/L in the WT/p.Asp410Ilefs*9 heterozygous variant, which had significantly decreased to 78 ± 0.1 and 58 ± 0.09 nmol/L of that of the WT, respectively. The concentration of DHEA was 0.72 ± 0.04 nmol/L in the p.Phe435Ser/p.Asp410Ilefs*9 compound heterozygous variant (**Figure 3D**).

These results indicated that the novel c.1304T > C and c.1228delG compound heterozygous variants of *CYP17A1* resulted in almost complete deficiencies of 17α-hydroxylase and 17,20-lyase activity, which were consistent with the blood hormonal characteristic of the proband.

## Discussion

In the present study, we investigated the clinical evaluation, genetic diagnosis, and molecular mechanisms underlying 17-OHD associated with a novel compound heterozygous *CYP17A1* variant. 17-OHD is an uncommon form of CAH. Here, an 11-year-old girl was characterized by 17-OHD with rhabdomyolysis and hypokalemia. Hypokalemic rhabdomyolysis has not been previously reported as a complication of 17-OHD except in the case of 11β-hydroxylase deficiency (Atabek et al., 2008; Yurekli et al., 2016). Severe hypokalemia usually results in capillary contraction and reduced muscle blood supply, leading to myocardial cell necrosis and rhabdomyolysis (Rossi et al., 2006; Huang et al., 2012; Marking et al., 2014). The patient’s clinical manifestation included hypokalemia, hypertension, and rhabdomyolysis. Her blood potassium level failed to recover *via* potassium supplementation. Glucocorticoid treatment began, and at follow-up, potassium levels had returned back to normal.

WES and Sanger sequencing identified the patient’s two variants of the *CYP17A1* gene associated with hypokalemia, hypertension, and rhabdomyolysis. The *CYP17A1* gene encodes 17α-hydroxylase/17,20-lyase (P450c17) (Krone and Arlt, 2009) and is highly expressed in the adrenal glands and gonads (Auchus, 2017). Approximately 129 different *CYP17A1* gene variants have been reported, including missense variants, small insertions or deletions, and splice site variants (http://www.hgmd.cf.ac.uk). Some *CYP17A1* variants, such as c.1216T > C/p.Trp406Arg, c.1247G > A/p.Arg416His, c.1250T > G/p.Phe417Cys, and c.1283C > T/p.Pro428Leu, are associated with complete 17-OHD. They grossly disrupt the coordination of the heme groups (Biason-Lauber et al., 2000; Martin et al., 2003; Ergun-Longmire et al., 2006). The novel c.1304T > C/p.Phe435Ser variant in this study may disturb the hydrophobic environment, thereby interfering with the catalytic activity and the heme-binding domain. There was no significant difference between the steady-state level of the p.Phe435Ser mutant and WT P450c17. The c.1228delG/p.Asp410Ilefs*9 deletion of *CYP17A1* resulted in the occurrence of a frameshift which led to a truncated protein. The protein level expression of the p.Asp410Ilefs*9 mutant was almost undetectable. Western blotting assays support the genetic analysis of this patient.

Furthermore, expression of p.Phe435Ser/p.Asp410Ilefs*9 P450c17 variants in HEK-293T cells showed loss of 17α-hydroxylase and 17,20-lyase activities, which was consistent with the clinical manifestations and hormonal characteristics of the proband (**Table 1**). Because 17,20-lyase catalyze 17-hydroxypregnenolone into DHEA, the deficiencies of 17,20-lyase decreased the level of DHEA and downstream products including androstenedione, testosterone, and estradiol. The patient’s sexual characteristics should be examined through karyotyping due to lack of the above sex steroids (Marsh and Auchus, 2014; Yang et al., 2019). On the other hand, 17α-hydroxylase catalyze progesterone into 17-OHP, and the deficiencies of 17α-hydroxylase led to the decrease of cortisol and a compensatory increase of 11-deoxycorticosterone (DOC). DOC excess causes hypertension and hypokalemia (Auchus, 2017; Sun et al., 2017; Alswailem et al., 2018). Complete deficiency of 17α-hydroxylase and 17,20-lyase activity could be used as a potential biomarker for future 17-OHD screenings.

In summary, a patient was characterized by 17-OHD with rhabdomyolysis, hypokalemia, and adrenal insufficiency. WES and Sanger sequencing identified a novel compound heterozygous *CYP17A1* variant associated with 17-OHD. The functional assays revealed that the compound heterozygous variant led to the deficiency of 17α-hydroxylase and 17,20-lyase activity. Our findings thus provide a novel insight into the clinical evaluations and molecular basis of 17-OHD.

## Ethics Statement

The study was reviewed and approved by the Ethics Committee of The First Affiliated Hospital, Zhejiang University, China (No. 2018-727), and was conducted in agreement with the Principles of the Declaration of Helsinki. Written informed consents were obtained from the patient’s parents.

## Author Contributions

QY, CW, and LL conceived and designed the research. HC, KY, BZ, ZJ, CC, YZ, YS, and HZ performed the experiments and analyzed the data. QY, HC, CW, LL, and WH wrote the manuscript.

## Funding

This work was supported by the National Basic Research Program of China (2014CB943001), National Natural Science Foundation of China (11571309, 31771398, and 31571299), Fundamental Research Funds for the Central Universities (2019QNA6001), Zhejiang Provincial Natural Science Foundation of China (LZ19C060001), and National Institute of Health (NCI 2R01CA139158).

## Conflict of Interest

The authors declare that the research was conducted in the absence of any commercial or financial relationships that could be construed as a potential conflict of interest.

## Supplementary Material

Figure S1 Karyotype of the proband: Normal 46,XX.

Figure S2 (A,B) Quality scores across all bases: An average base quality of 40 in FASTQ fles and a coverage of 99.59% in targeted regions.

Figure S3 Conservative analysis in different vertebrates: Phe435 is conserved among the P450c17s from different vertebrates (Pan troglodytes, Felis catus, Mus musculus, Gallus gallus, Danio rerio, and Xenopus tropicalis).

Figure S4 Quantitative PCR results: There are no CYP17A1 gene differences in mRNA levels among groups.
