# Supplementary figures and images for: A Novel Compound Heterozygous CYP17A1 Variant Causes 17α-Hydroxylase/17, 20-Lyase Deficiency
Source: Front Genet. 2019 Oct 22;10:996. doi: 10.3389/fgene.2019.00996 (PMC6817513; doi:10.3389/fgene.2019.00996)

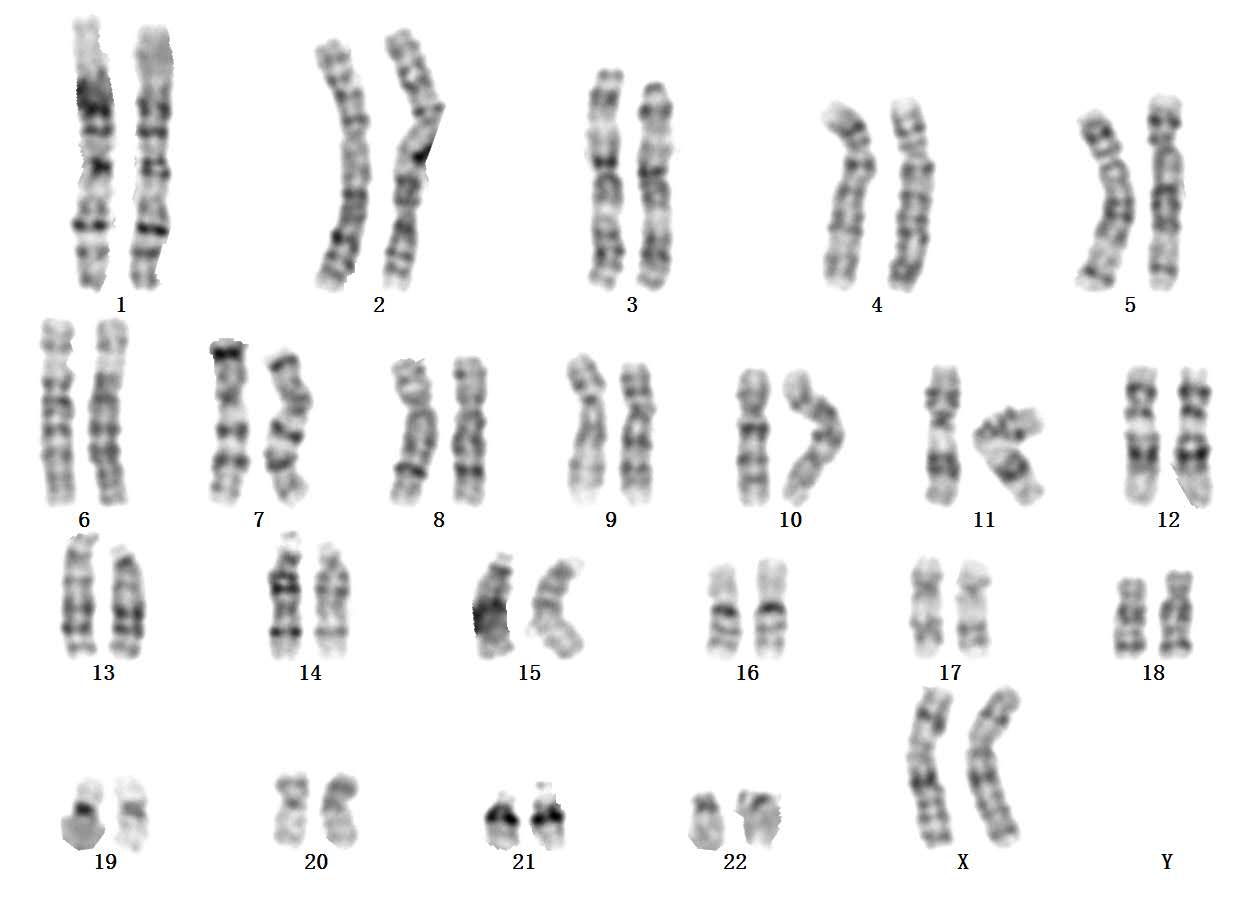

Supplement: Figure S1 — Karyotype of the proband: Normal 46,XX. [file Image_1.jpeg]

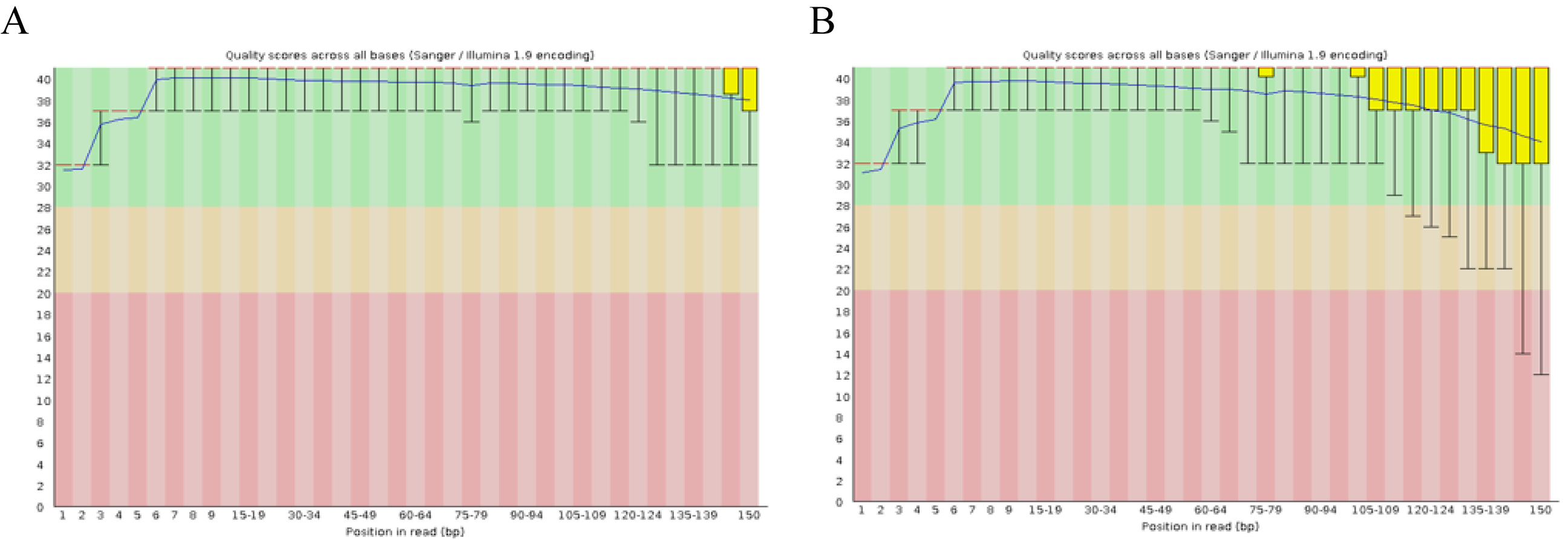

Supplement: Figure S2 — (A,B) Quality scores across all bases: An average base quality of 40 in FASTQ fles and a coverage of 99.59% in targeted regions. [file Image_2.tif]

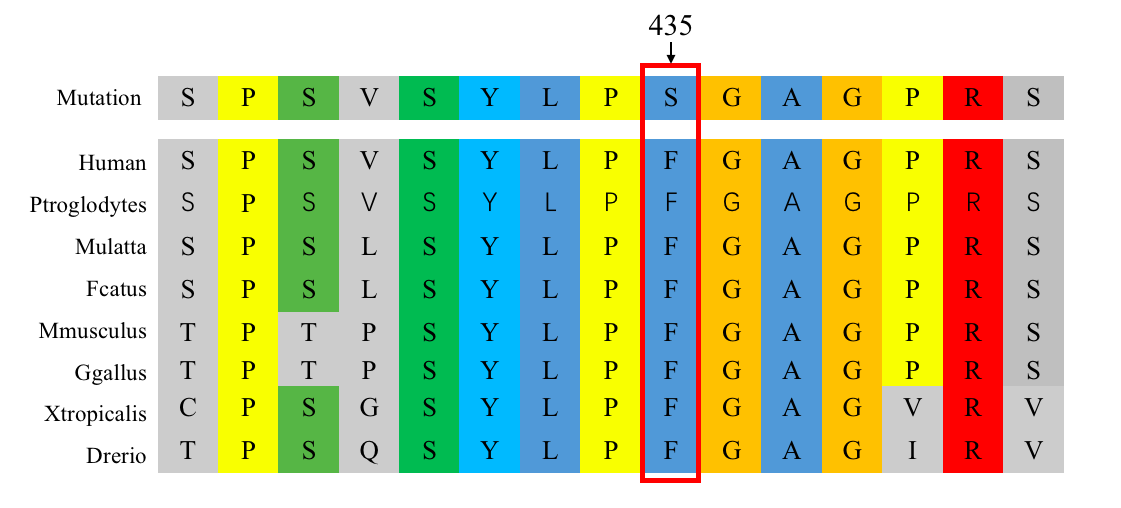

Supplement: Figure S3 — Conservative analysis in different vertebrates: Phe435 is conserved among the P450c17s from different vertebrates (Pan troglodytes, Felis catus, Mus musculus, Gallus gallus, Danio rerio, and Xenopus tropicalis). [file Image_3.tif]

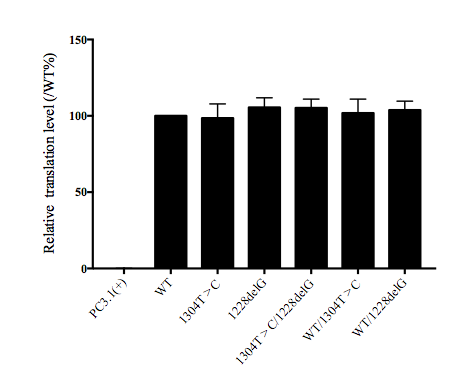

Supplement: Figure S4 — Quantitative PCR results: There are no CYP17A1 gene differences in mRNA levels among groups. [file Image_4.tif]
