# Supplementary material for: A Novel Compound Heterozygous CYP17A1 Variant Causes 17α-Hydroxylase/17, 20-Lyase Deficiency
Source: Front Genet. 2019 Oct 22;10:996. doi: 10.3389/fgene.2019.00996 (PMC6817513; doi:10.3389/fgene.2019.00996)
Supplement: Supplementary file 5 [file Table_1.docx]

Table S1. Mutagenic primers

| Variants |  | Sequence (5’→3’) |
| --- | --- | --- |
| c.1304T>C | Forward | GGAGTGGCACCAGCCGATCAGTTCATGCCTGAGCGT |
|  | Reverse | AGACAGATCGCTGAGATAGGTGC |
|  | Forward | CGGCTGGTGCCACTCCTTCT |
|  | Reverse | AGGCACCTATCTCAGCGATCTG |
| c.1228delG | Forward | TCAGTAAGCTATTTGCCCTGCGGAGCAGGACCTC |
|  | Reverse | AGACAGATCGCTGAGATAGGTGC |
|  | Forward | AGGTCCTGCTCCGCAGGGCAAATAGCTTACTG |
|  | Reverse | AGGCACCTATCTCAGCGATCTG |
