# Supplementary material for: A Novel Compound Heterozygous CYP17A1 Variant Causes 17α-Hydroxylase/17, 20-Lyase Deficiency
Source: Front Genet. 2019 Oct 22;10:996. doi: 10.3389/fgene.2019.00996 (PMC6817513; doi:10.3389/fgene.2019.00996)
Supplement: Supplementary file 6 [file Table_2.docx]

Table S2. Summary of original whole exome sequencing data.

| Data | Proband |
| --- | --- |
| Number of raw reads (M) | 82.90 |
| Number of reads mapped to the genome (M) | 82.79 |
| Average read length (bp) | 150 |
| Fraction of uniquely mapped bases on target (%) | 58.59% |
| Mean Mapping Quality | 58.35 |
| Mean depth of target region (fold) | 142.90 |
| Coverage of target region (%) | 99.59% |
| Target region with more than 30X (%) | 98.37% |
